# Experimental based experiences with the introduction of a water safety plan for a multi-located university clinic and its efficacy according to WHO recommendations

**DOI:** 10.1186/1471-2458-7-34

**Published:** 2007-03-13

**Authors:** Alexander Dyck, Martin Exner, Axel Kramer

**Affiliations:** 1 Institute for Hygiene and Environmental Medicine of the Ernst-Moritz-Arndt-University, Walther-Rathenau Straße 49A, 17489 Greifswald, Germany; 2 WHO Collaborating Centre for Health Promoting Water Management and Risk Communication at the Institute of Hygiene and Public Health, Sigmund-Freud-Str. 25, 53105 Bonn, Germany

## Abstract

**Background:**

Due to the high number of immunosuppressed and other predisposed patients hospitals have to control and ensure the microbiological water quality. The origin for the occurrence of pathogenic microorganisms in water pipes is the formation of biofilm.

**Methods:**

For the permanent control of water safety a water safety plan (WSP) was realized as recommended by the WHO following the principle "search and destroy". The WSP is based on an established HACCP concept due to the special focus. The most important measures include the concept for sample taking depending on patient risk. 3 different categories) are distinguished: risk area1 (high infection risk), risk 2 (moderate infection risk), and risk area 3 (not increased infection risk). Additionally to the threshold value of the German law for the quality of drinking water (TrinkwV) three more limiting values were defined (warning, alert, and worst case) for immediate risk adapted reaction. Additional attention has to be focussed on lavatory sinks, which are an open bacterial reservoir. Therefore continuous disinfecting siphons were installed as part of the WSP in high risk areas.

If extended technical equipment is not available, especially for immunocompromised patients the following measures are easy to realize: boiled (or sun exposed) water for nursing procedures as well alimentary use, no showering.

**Results:**

Comparing data over 3 years the microbial water quality was significantly improved resulting in no new case of nosocomial Legionella pneumoniae and decrease in neonatal sepsis.

**Conclusion:**

According to average situations with highly contaminated water system the management must be defined with implementation of water task force, immediate providing of special equipment, information of patients and staff and control of the water quality, an example for successful decontamination of the hospital within 24 hours is given.

## Background

The coherence between contaminated water and nosocomial infections are still a common problem in the clinical routine [1]. The dominating pathogens are *Legionella pneumophila *[2] and *Pseudomonas aeruginosa *[3], while i.e. *Aeromonas spp*., *Flavobacterium spp*., atypical Mycobacteria (Mycobacteria other than tuberculosis = MOTT) [4,5], *H. pylori *[6], Acanthamoeba [7], Cryptosporidia [8], Amoebae i.e. Naegleria [9], Viruses [10], Shigella [9], *Salmonella spp*., EHEC and moulds like *Aspergillus fumigatus *[11,12] and fusarium [13] are found less frequently. There are additionally epidemic agents in developing countries like *Vibrio cholerae*, *Entamoeba histolytica *and *Salmonella typhi *[9].

Due to the high number of immunosuppressed or other endangered patients in hospitals, the requirements on the microbiological quality of the drinking water are much higher than in domestic area. The occurrence of pathogenic microorganisms in water pipes is caused by biofilms [1,14,15]. These biofilms arise not only in older but also in newly opened hospitals mostly due to stagnation [16]. Further attention has to be focussed on lavatory sinks, containing up to 10^5 ^to 10^10 ^cfu/ml of bacteria, thereof about 10^3 ^to 10^6 ^cfu/ml of gramnegative rods [17].

For prevention of nosocomial waterborne infections a structured quality management and sufficiently operating security system has to be established. In 2004, the WHO published the 3^rd ^guidelines for drinking water quality recommending the introduction of a water safety plan (WSP). It has to include control and preventive measures, based on a multiple-barrier approach and the HACCP (hazard analysis and critical control points) concept [18]. The permanent surveillance of the microbiological water quality as well as the realization of the WHO guidelines is the aim of our efforts for the introduction and evaluation of the WSP for a hospital of maximum care.

## Methods

### Realization of the HACCP concept in routine

**Figure 1:**
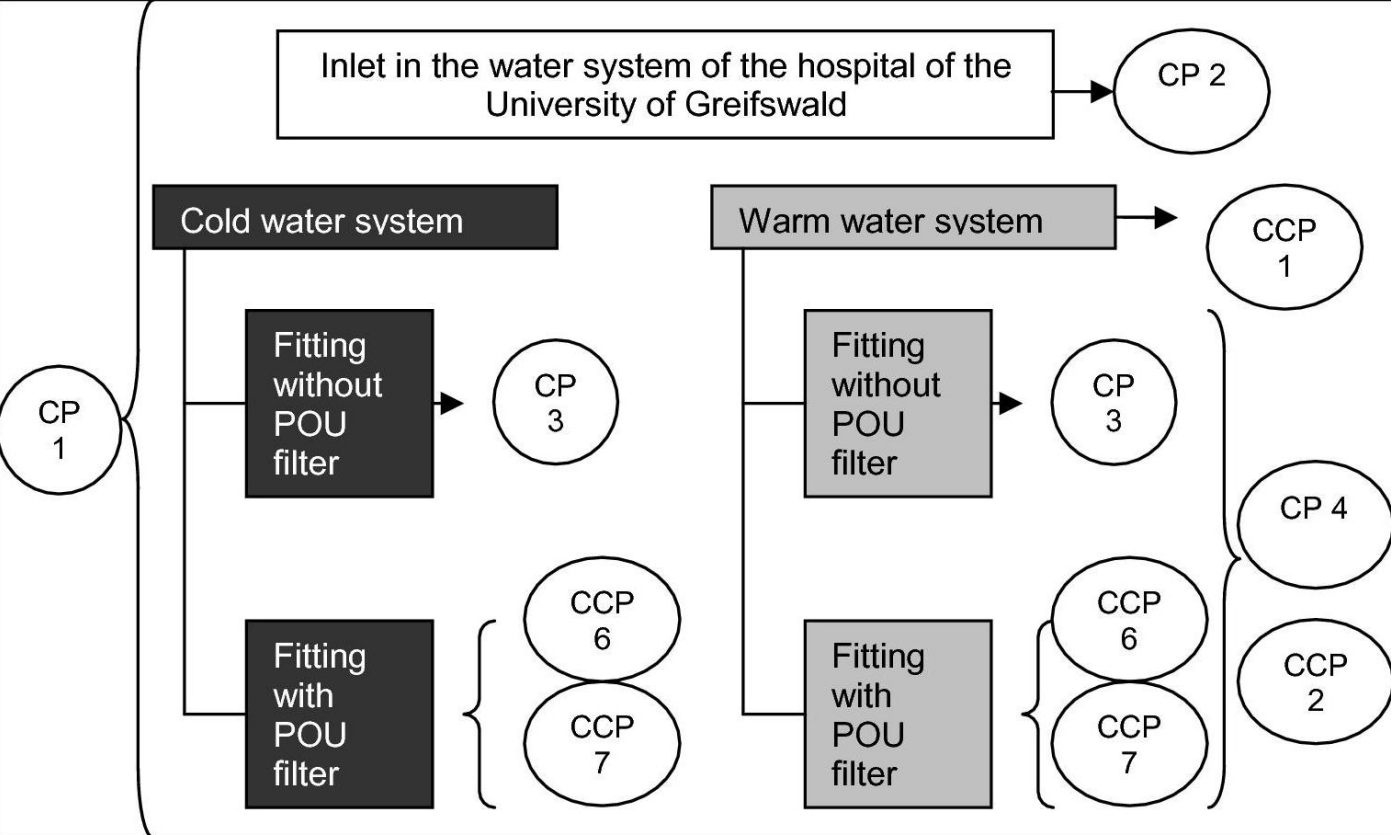
CP and CCP in routine.

**Figure 2:**
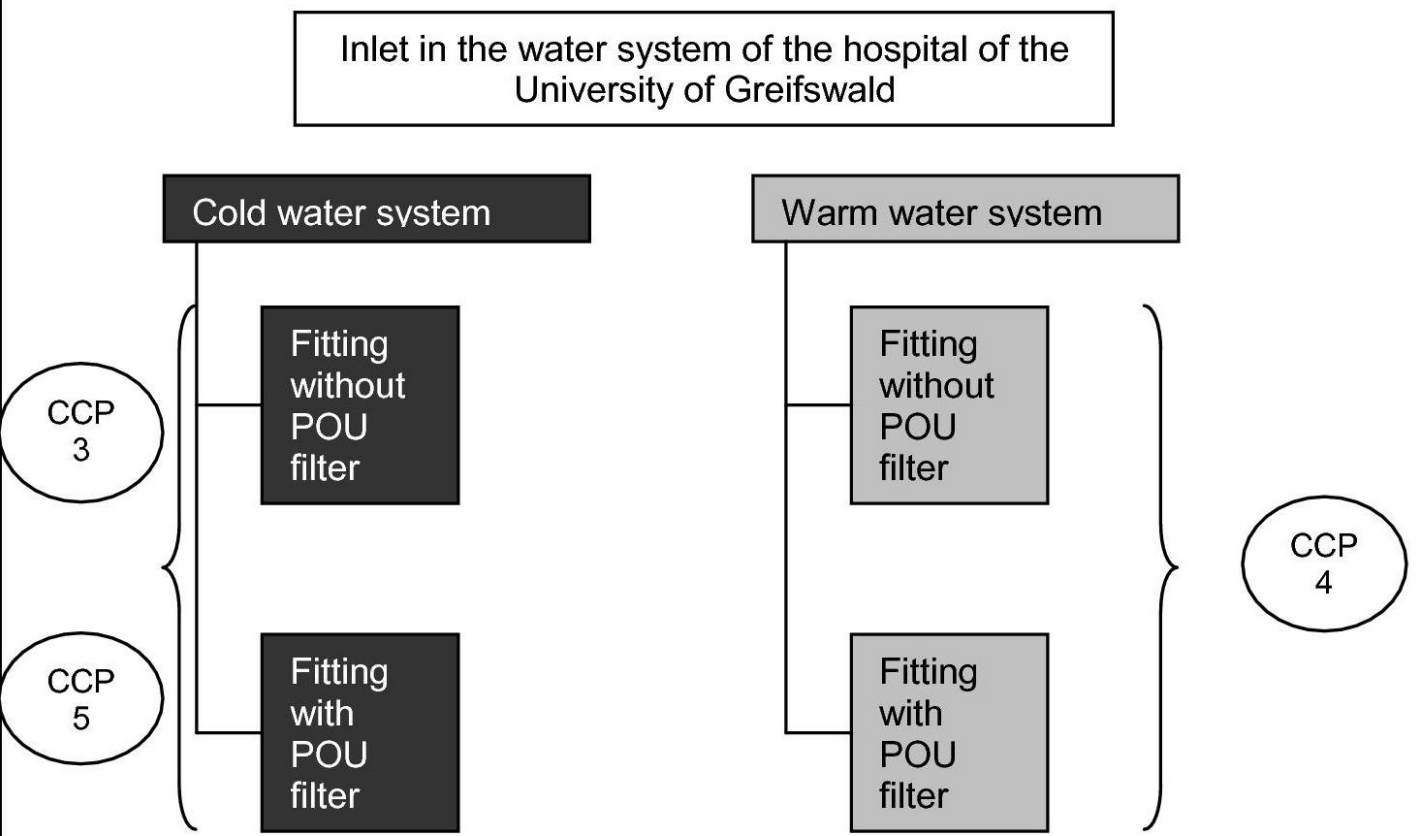
CP and CCP during the process of decontamination.

CCP and CP were introduced by identification of microbiological, chemical and physical risks and the definition of concrete points of threat (control points; at least every fact which has an influence on the water quality) and points of steering (critical control points; threats which offer the possibility of exerting influencing control and avoidance of risks). Both are considered under two situations: routine (figure 1) and critical contamination (figure 2).

The following critical control points (CCP) were defined:

• CCP 1 incoming temperature in the warm water system at least ≥ 60°C

• CCP 2 monthly thermal disinfection of the warm water system at a temperature of 73°C for at least 10 min

• CCP 3 concentration of disinfecting agents for the chemical disinfection at point of entrance and taps; limiting values according to the German law for the quality of drinking water (TrinkwV 2001) have to be maintained

• CCP 4 compliance of the temperature-time relation according to thermal disinfection in case of positive Legionella results in the water

• CCP 5 compliance of limiting values after chemical decontamination at the outlets

• CCP 6 changing frequency of point of use (POU) filters

• CCP 7 surveillance of the processing of POU filters

The following control points (CP) were detected:

• CP 1 transgression of microbiological limiting values

• CP 2 water inlet in the pipeline system of the hospital

• CP 3 handling and processing of the aerators

• CP 4 scalding risk at thermal disinfection including outlets

As a result of the definition of CP and CCP the redevelopment and basic decontamination of the water pipe system was the first preventive measure. Rarely used taps and dead ends were detected and removed. Concrete measures to be performed in case of exceeding 80% of the legal defined limit value of 100 cfu/ml were predetermined (so-called warning value, table 1). The intention behind this sub-set limit value is the prevention of critical contamination.

**Table 1:** Definition of extended microbiological thresholds according to the risk areas

**Risk area**	**Parameter**	Warning*	**Threshold**	Alert*	Worst case*
**1 and 2**	TMC 22°C and 36°C	80 – 100 CFU/ml	>100 CFU/ml**	>200 – 500 CFU/ml	>500 CFU/ml
	Coli-like, *E. coli*, Enterococci		0 CFU/100 ml**	1–10 CFU/100 ml	>10 CFU/100 ml
	*P. aeruginosa*		0 CFU/100 ml**	1–10 CFU/100 ml	>10 CFU/100 ml
	*Legionella spp.*(shower, tap)		0 CFU/100 ml*	1–100 CFU/100 ml	>100 CFU/100 ml
	fungi		0 CFU/100 ml*	10–100 CFU/100 ml	>100 CFU/100 ml
**3**	TMC 22°C and 36°C		>100 CFU/ml**	>500 CFU/ml	>1000 CFU/ml
	Coli-like, *E. coli *Enterococci		0 CFU/100 ml**	1–10 CFU/100 ml	>10 CFU/100 ml
	*P. aeruginosa*		0 CFU/100 ml**	1–50 CFU/100 ml	>50 CFU/100 ml
	*Legionella spp.*(shower)		0 CFU/100 ml*	>5–50 CFU/100 ml	>50 CFU/100 ml
	*Legionella spp. *(tap)		0 CFU/100 ml*	>200 CFU/100 ml	>500 CFU/100 ml

*our in-house recommendation ** law(2001) resp. water quideline (2004)

The following measures were established:

• ≥ 80 cfu/ml in the immediate sample after opening the tap as sign for local contamination, but no pathogens: processing of the aerators

• cfu ≥ 80 cfu/ml after 3 min of running as indicator for central contamination, but no pathogens: flooding of water system, control, if still positive (> 100 cfu/ml), ClO_2 _decontamination (6 ppm 1 h)

• *Pseudomonas spp. *in 100 ml: temporarily installation of POU filters in risk areas until decontamination

• *Legionella spp. *in 100 ml: POU resp. closing of showers until decontamination, permanent heating level of 60°C in the hot water system, additionally monthly thermo disinfection of hot water system including all outlets permanent installation of point of use (POU) filters in risk wards as well as for the last washing cycle for endoscope instruments. The heating up minimal to 73°C for at least 10 min is necessary for killing intra-amoeboid Legionella [19]. In case of contamination of the cold water system the same procedure is realized.

### Realization of the HACCP concept in case of water stagnation (i.e. reconstructed or new buildings)

In case of stagnation, i.e. during times of reconstruction of elder or new building under construction the following measures are executed:

• Flushing of the water system beginning 4 weeks before opening

• Parallel water sampling

• If flushing is not sufficient; decontamination depending on the microbial burden and the risk assessment

### Realization of the HACCP in case of emergency

The task force water safety releases immediate measures in case of contamination:

• No withdrawal of water until the end of decontamination

• Avoid hand washing as far as possible; prefer disinfection

• If hand washing is necessary, disinfect the hands subsequently, or use filtrated water (POU filter)

• Use heated water (≥ 73°C 10 min) for washing the patients

• Showering is forbidden during the hole process of sanitation

• Water for brushing teeth has to be taken from fountains or mineral water bottles

• Contaminated water may not be used for cleaning surfaces near to patients

• All outlets in working areas have to be equipped with POU filters.

The process of decontamination is executed during the night shift. Staffs needs to be informed about the measures, every ward has a responsible employee. The first step is the de-installation and processing of all aerators. Afterwards the hot water system is heated up to a temperature of ≥ 73°C for at least 10 min, starting when achieving target temperature at the most distant outlet. The cold water system is decontaminated with ClO_2_(10 ppm). The concentration is checked at the most distant outlet just as in the hot water system. When achieving the default concentration, the acting time of 1 h begins. Afterwards the loss of chlorine is determined, if loss is more than 50 %, the process needs to be repeated. The whole water system is flushed until a concentration of ≤ 0.2 ppm ClO_2 _is attained. Machines, like drinks dispenser, with connection to the drinking water system are disconnected before.

### Implementation of a microbiological control program and predetermined measures

According to the German drinking water ordinance (TrinkwV 2001) the drinking water must be examined for the indicator organisms Coli-like, *E. coli*, Heterotrophic plate count (HPC) in cold water at 22°C and 36°C and *Legionella spp. *in water > 21°C once a year. Further controls were introduced basing on risk assessment: Risk area 1 (high infectious risk), risk area 2 (moderate infectious risk), risk area 3 (infectious risk not increased). The frequency of controls is 3, 2 resp. 1 per year (table 2).

**Table 2:** Allocation of the different facilities according to the risk assessment

**Risk area**	**Clinic**	**Ward/Department**
**1 POU filter**	anaesthesia and intensive medicine	ICU
	gynecology	delivery room-birth tub
	otorhinolaryngology	water for processing of endoscops
	internal medicine	ICU, hematology-oncology
	pediatrics	milk kitchen, Hematology/Oncology, ICU, Processing of incubators
**2**	anaesthesia and intensive medicine	weaning
	surgery	intermediate care
	gynecology	senology-breast center
	otorhinolaryngology	radiol. ward
	internal medicine	cardiologic monitoring ward
	oral and maxillofacial surgery	Recovery room
	nuclear medicine	ward
	neurorehabilitation	all wards/divisions
**3**	anaesthesia and intensive medicine	stroke unit
	ophtalmology	all wards
	surgery, gynecology, dermatology otorhinolaryngology	all other wards
	internal medicine	dialysis, nephrology, gastroenterology, endocrinology, admission ward, cardiology, emergency
	pediatric surgery, oral and maxillofacial surgery, pediatrics, neurosurgery neurology, orthopedics, urology	all other wards
	center for dentistry	all divisions
	hospice	ward

SOP's were introduced for water sampling and processing of the samples steered by a sampling plan. The sampling is performed immediately after opening the tap and after 3 min running. The microbiological diagnostic follows national recommendations [20-24], while Legionella is determined in 1000 ml water instead of 100 ml recommended by the Federal Environmental Office (Umweltbundesamt)

In risk area 1 permanent pathogen-free water is required. Because permanent supervision is impossible single-use or reprocessable point of use (POU filters) are provided. Filters are automatically reprocessed in a washer disinfector at the certified university-own Central Sterile Supply Department and finally dryed at 115°C with sterile filtrated air. For safe handling (touching and cleaning is prohibited, changing frequence is 4 resp. 8 weeks) a hygienic information sheet was deposited at all places of usage.

Additionally the self disinfecting siphons BioRec^® ^(BIOREC UmweltBioTechnologie& BioRecycling-Systeme Lauta, Germany), which acting by self-heating, antimicrobial coated, and emitting ultrasound, to prevent the emission of aerosols [17], were installed in risk area 1.

The extended limiting values beside the limiting value (according to the TrinkwV) are the warning value, the alert, and worst case value, based on a risk assessment (table 1).

Table 3 gives an overview measures that were established.

**Table 3:** the different schemes in case of exceeding microbiological limit values for risk and non-risk areas

**Risk area**	**Parameter**	**Warning**	**Threshold**	**Alert**	**Worst case**
**1**	POU filter				
**2**	CFU/1 ml 22°C, 36°C	inspection,	inspection, flushing, POU filter,	meeting of taskforce water safety,	meeting of taskforce water safety, stop of withdrawal,
		flushing,	control within 1 day,	Inspection,	disinfection,
		control within 3 days	If negative → finish use of POU filter	Flushing,	
			If still positive → disinfection	disinfection, POU filter, control within 1 day	
	P. aeruginosa,		inspection, POU filter,		meeting of task force water safety,
	Legionella spp.,		flushing, control within 1 d,		stop of withdrawal or POU filter, disinfection,
	E. coli Enterococci,		if negative → finish use of POU filter		control within 1 day,
	fungi		if still positive → disinfection		If negative → withdrawal allowed with POU for 5d,
					parallel water sampling
**3**	CFU/1 ml 22°C, 36°C		inspection, flushing, control within 3 days,		meeting of task force water safety, flushing,
			If still positive → disinfection,		disinfection,
			flushing,		control within 1 day
			control within 7 d		
	P. aeruginosa,		inspection,		meeting of task force water safety,
	Legionella spp.,		POU filter, flushing, control within 1 day, if negative → finish POU usage		stop of withdrawal or POU filter,
	E. coli,		if still positive → disinfection		if negative →
	Enterococci,				release of withdrawal and POU for 5d, parallel water sampling
	fungi				

The documentation for the WSP includes information about the ward, the isolated strain and content of cfu/ml, characteristics of the location (i.e. cold or warm water system; shower, tap) and the date of sample taking and provides information about performed measures (table 3).

### Formation of the task force "water safety"

To ensure the interdisciplinary cooperation of all responsible persons the task force water safety was introduced under direction of the head of the Institute of Hygiene and Environmental Medicine (IHEM), including two infection control nurses, experts of the institute of microbiology, hospital hygiene-supervising physicians from the different facilities and the department of engineering. The department of health of the city of Greifswald (DHC) supports our measures as external quality control authority.

## Results

In 2004 very high concentrations of micro-organisms occurred in a new opened part of the hospital. The department of health (DHC) had declared negative water probes within three month before the opening. Own controls at the day of official opening showed *P. stutzeri *at a concentration of 2.3 × 10^5 ^cfu/100 ml. The task force water safety initiated all measures now established in the WSP. The whole process of decontamination lasted at least 6 hours and took place during the night shift. All aerators were de-installed, processed and disinfected; the cold water system was decontaminated with 10 ppm ClO_2 _for one hour, followed by 0.2 ppm ClO_2 _over one week. The hot water system was heated up to 73°C for minimum 10 minutes. Water samples after sanitation until today indicated no further contamination.

**Figure 3:**
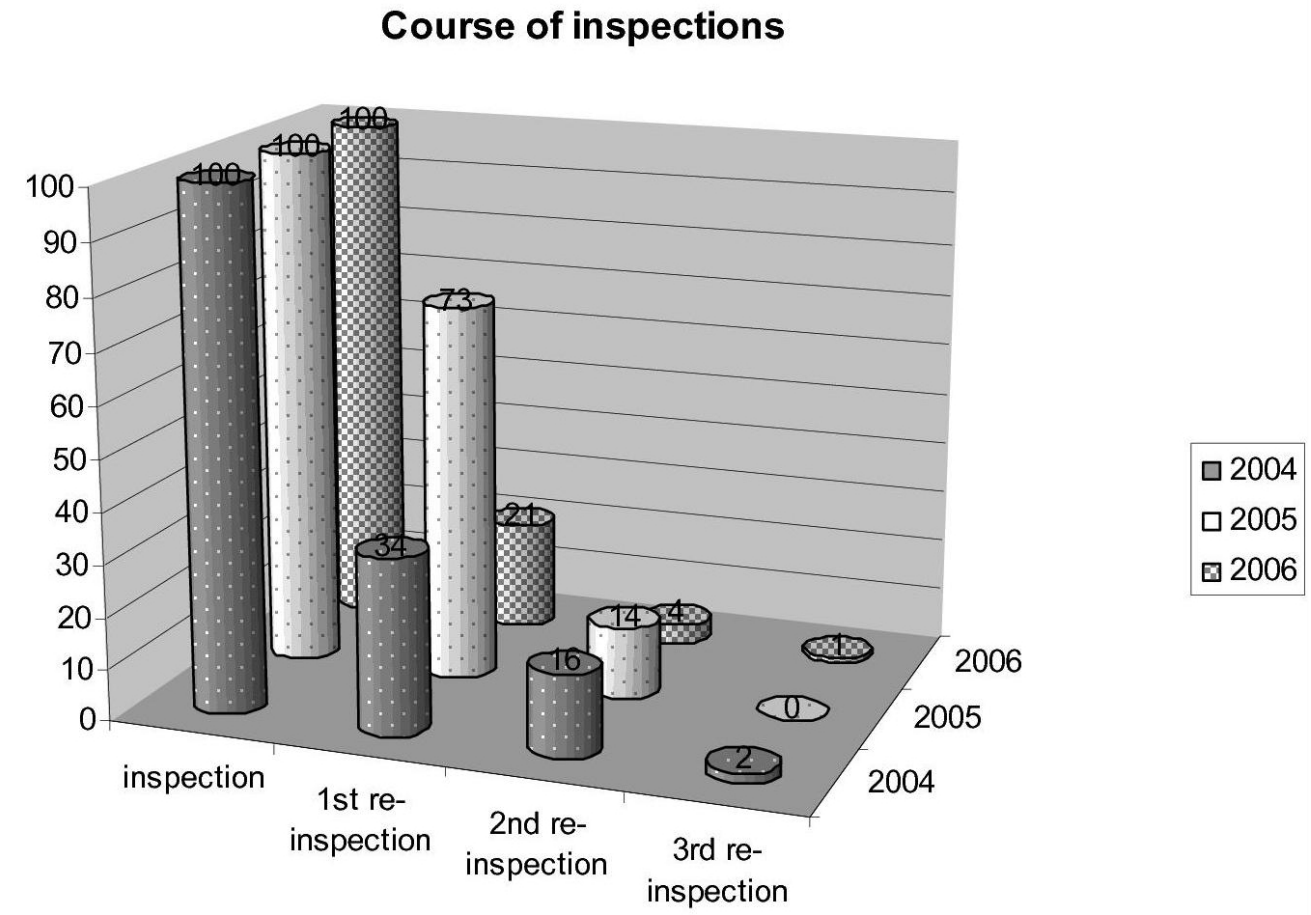
Course of the number of inspections by the IHU comparing the three years of the survey.

To evaluate the efficacy of the WSP the microbiological results were recorded from May 2004 to April 2006. Figure 3 shows the number of examinations (by means of comparability of ratios inspections are normalized to 100). The total numbers for 2004 were 56 inspections, 19 1^st^, 9 2^nd ^and 1 3^rd ^re-inspection, in 2005 respectively 78 inspections, 57 1^st^, 11 2^nd ^and no 3^rd ^re-inspection and 2006 finally 94 inspections, 20 1^st^, 4 2^nd ^and 1 3^rd ^re-inspection.

**Figure 4:**
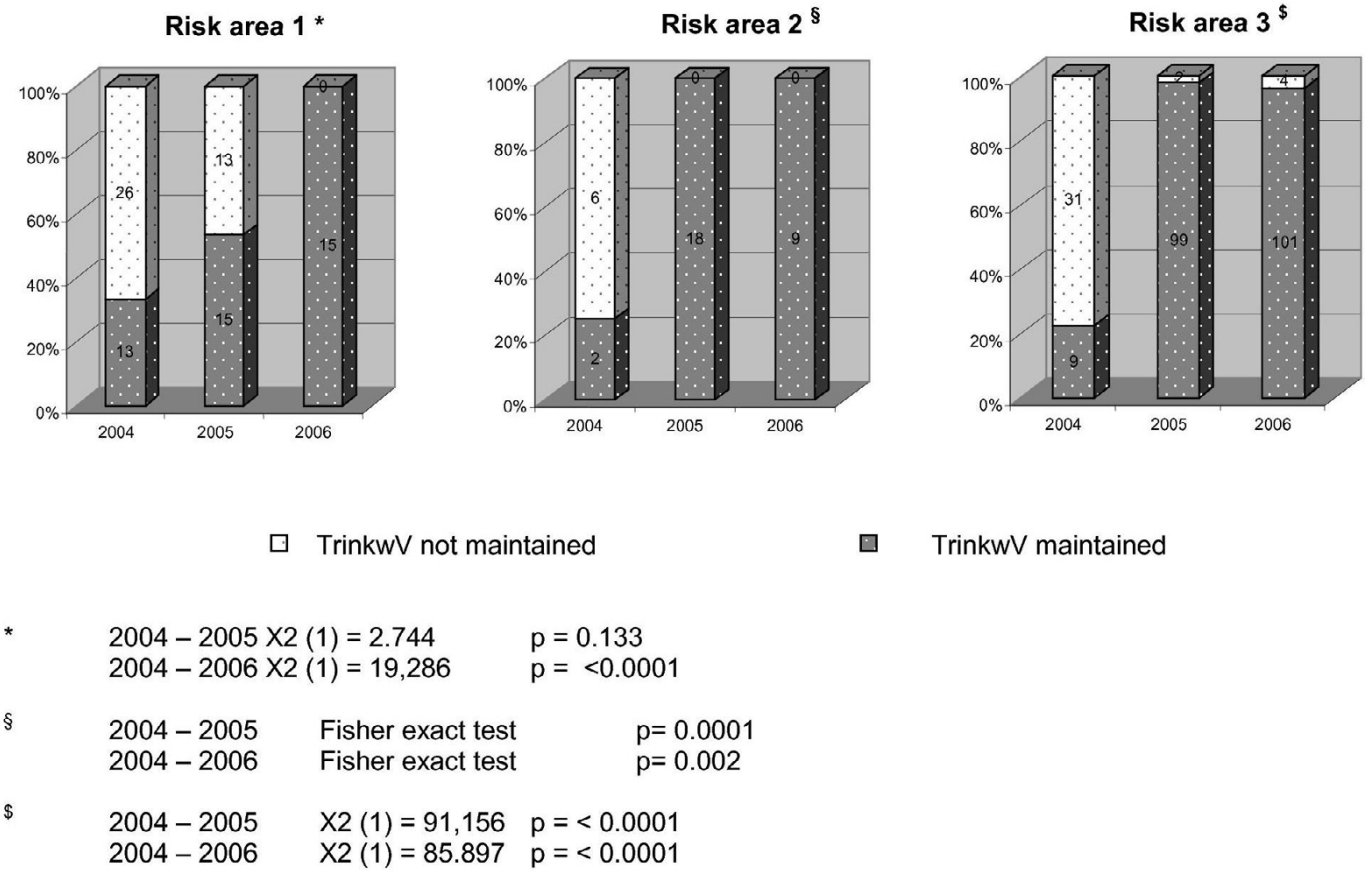
Development of the compliance of results according to the limiting values of the TrinkwV in the three risk areas (surveillance of IHEM).

**Figure 5:**
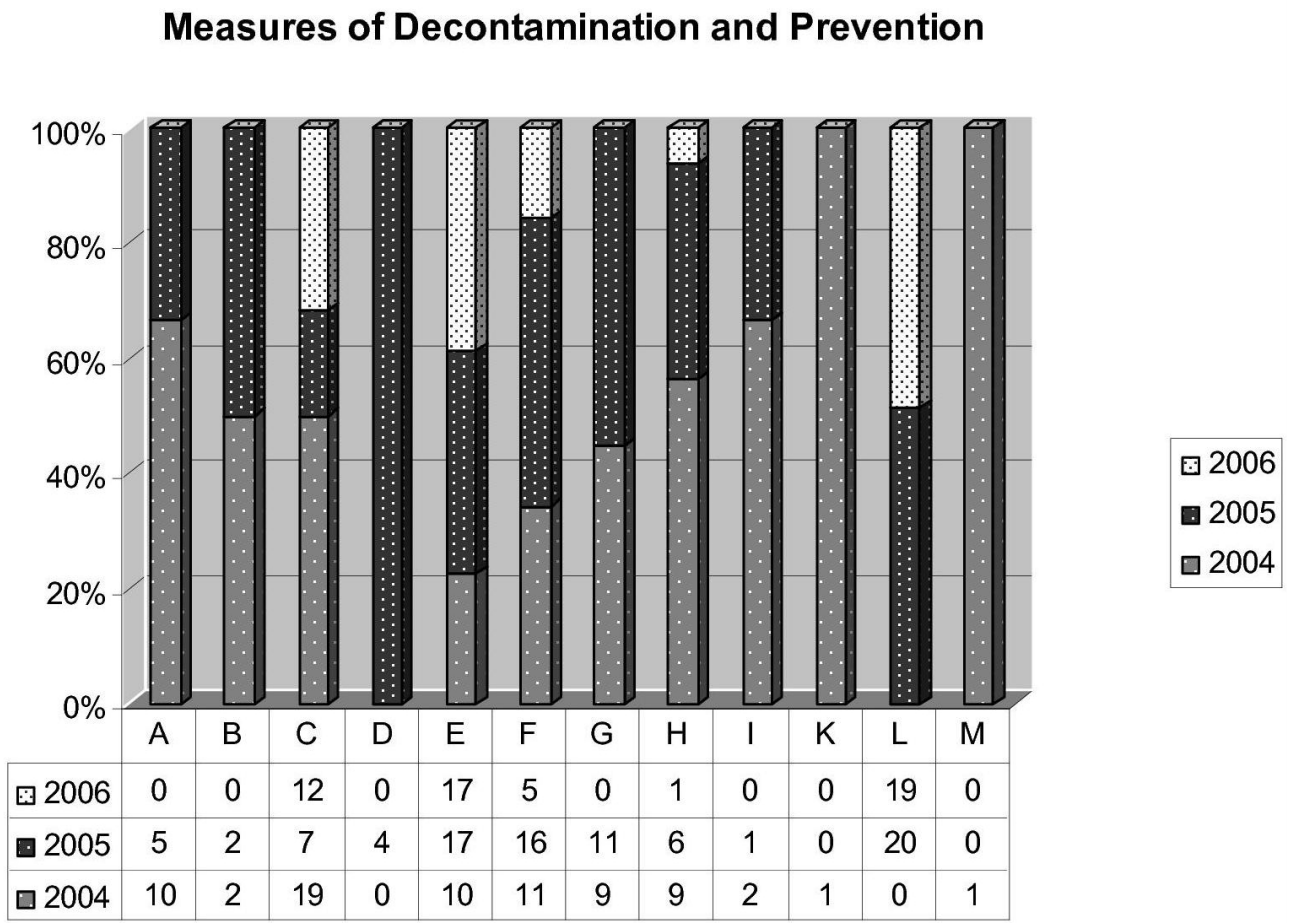
Measures executed for preventive and reasons of decontamination over the period from 2004 till 2006 (surveillance of IHEM). A = Immediate re-inspection; B = Re-inspection within 7 days; C = Re-inspection within 14 days; D = Re-inspection without POU filter; E = Re-inspection/processing of aerators; F = Flushing; G = ClO_2_-decontamination; H = Installation of POU filter; I = POU reversal; K = POU remains; L = Heating up; M = Closure of tap

Figure 4 gives an overview about performed measures; Figure 5 the maintenance of limiting values.

**Figure 6:**
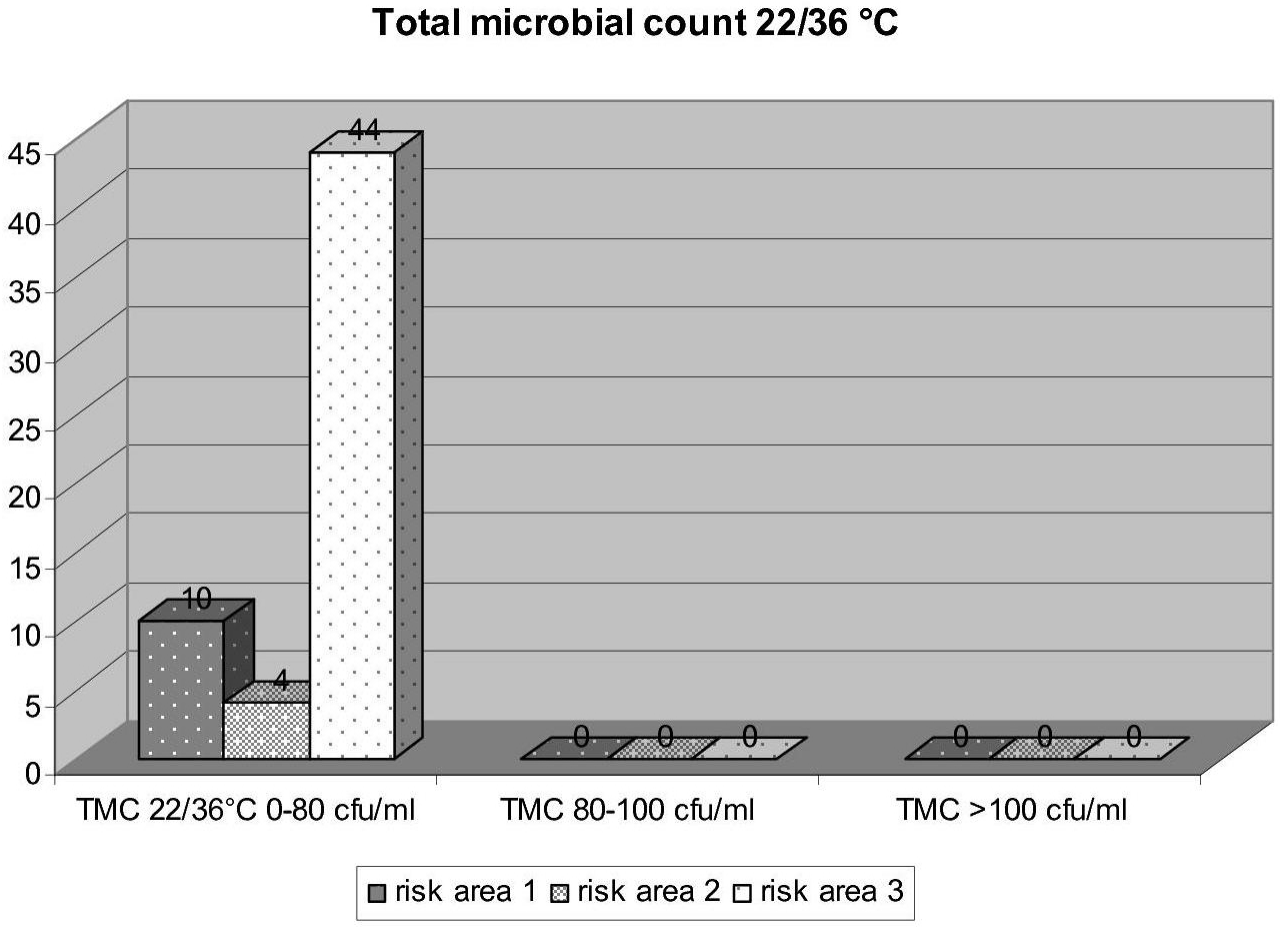
Results of examinations for heterotrophic plate counts/ml in the three risk areas (DHC 2005).

**Figure 7:**
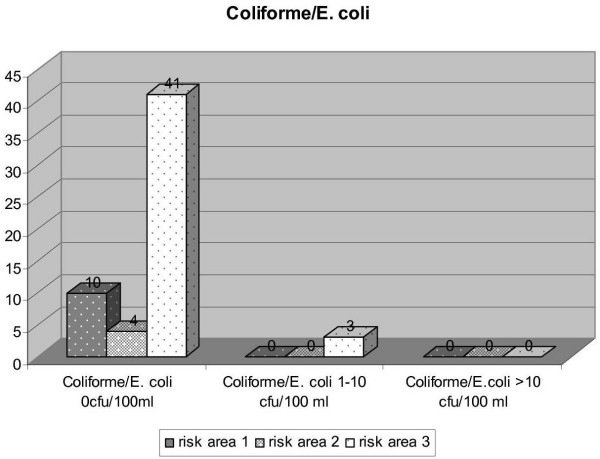
Results of examinations for Coli-like resp. *E. coli *in the three risk areas (DHC 2005).

**Figure 8:**
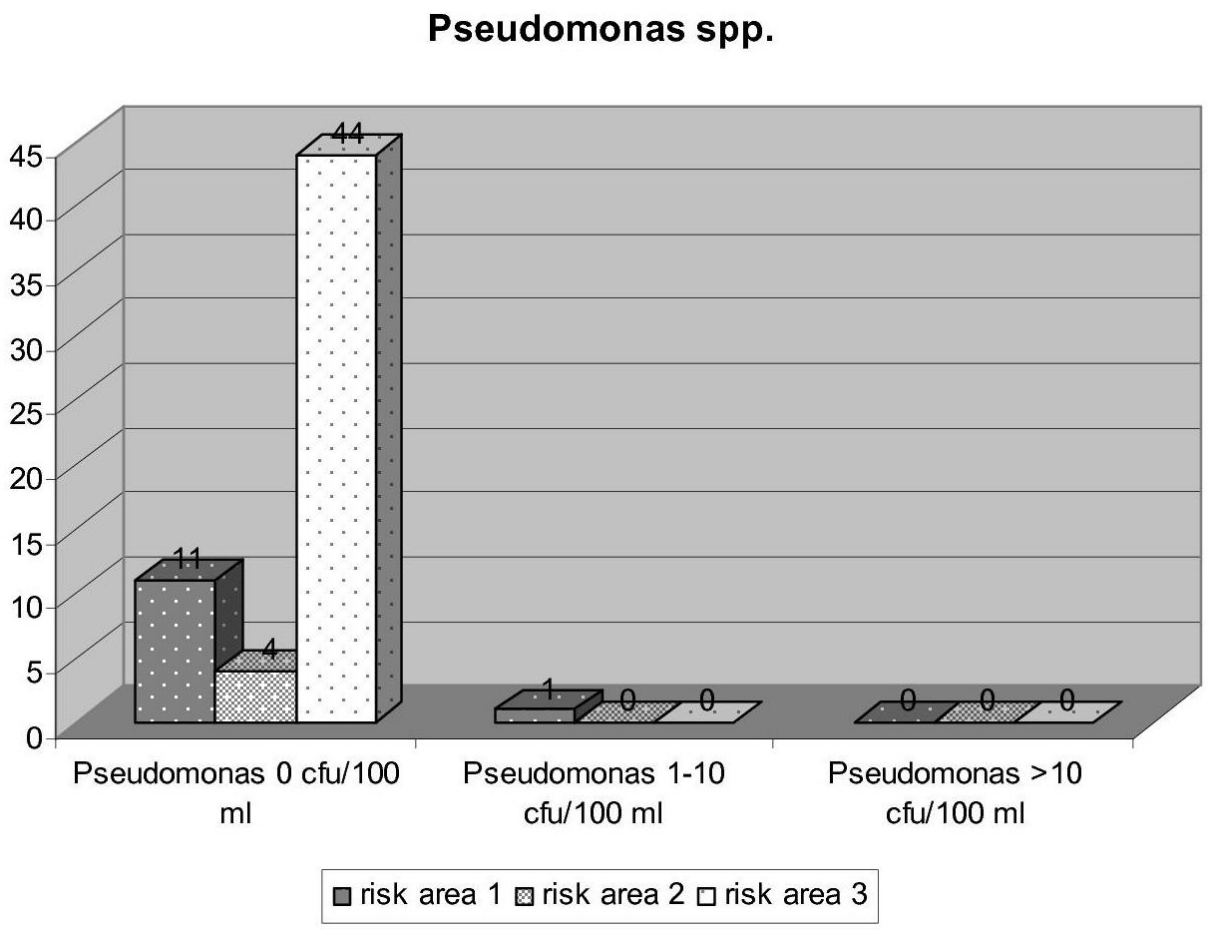
Results of examinations for *Pseudomonas spp. *in the three risk areas (DHC 2005).

**Figure 9:**
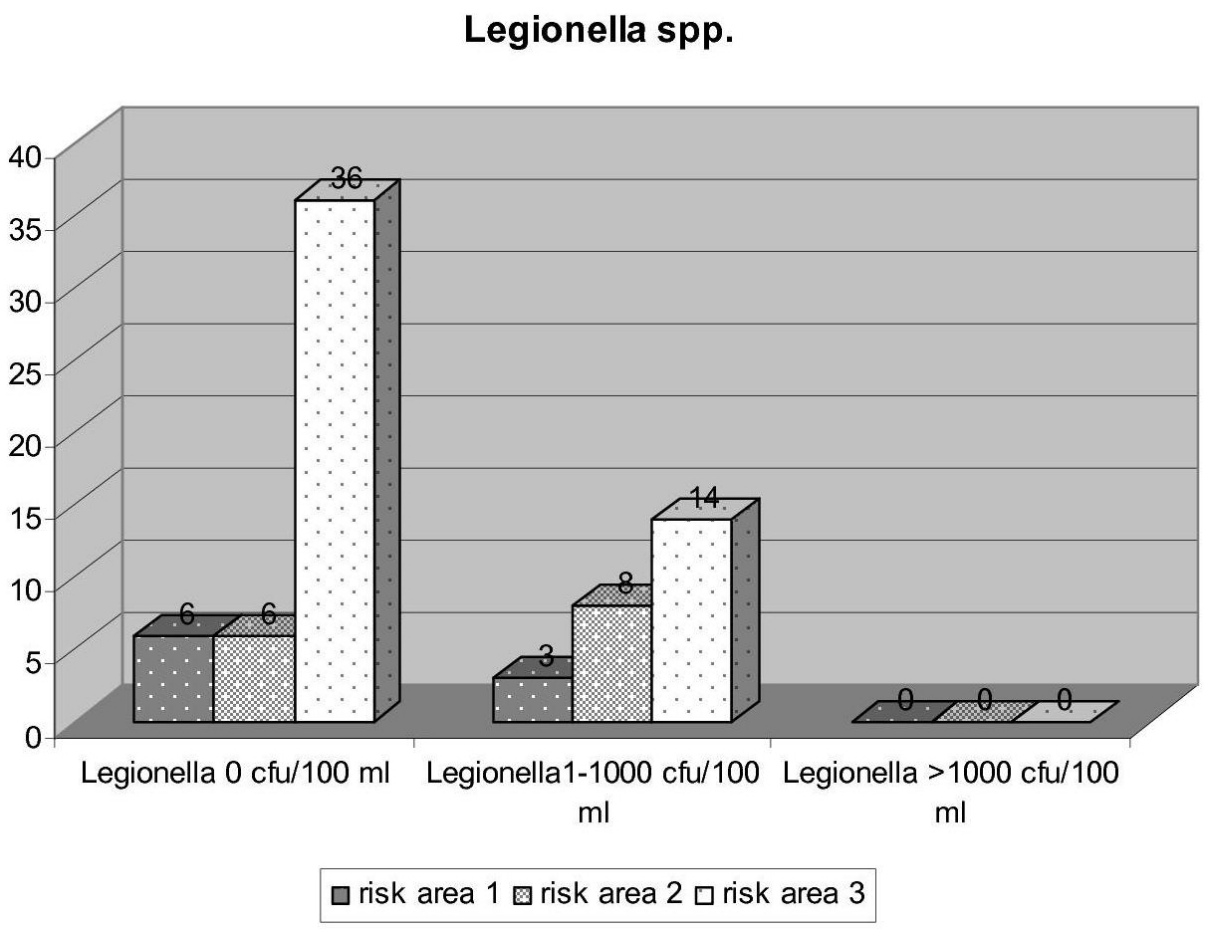
Results of examinations for *Legionella spp. *in the three risk areas (DHC 2005).

To demonstrate the outcome of the performed measures inspections by the DHC in 2005 provide a basis for considering their outcome. In total 21 inspections were performed in risk area 1, 28 in risk area 2 and 89 in risk area 3. Figure 6 to 9 express the microbiological results of the examinations by the DHC. There were only few complaints, mostly due to local contamination.

## Discussion

The first step for the introduction of a WSP is the revision of infrastructural failures regarding the possibility of a certain contamination. Therefore the infrastructure of the water system was analyzed and dead ends and rarely used taps were eliminated as far as possible in the elder parts of the hospital. These experiences were also adopted for the design of buildings now under construction. Those technical respectively infrastructural failures can result in extensive additional costs or even temporarily closure of parts of the clinical complex. Furthermore there are lots of other infrastructural conditions affecting the water quality. Leprat et al. [25] identified i.e. non-touch fittings as another special source of *Pseudomonas aeruginosa *and *Legionella spp*. We experienced wetting of those fittings during construction followed by stagnation as main reason of contamination. Therefore press-fittings now have to be moistened with water of proven drinking water quality to avoid a contamination.

To permanently ensure the water quality in high-risk areas terminal POU filters were established in risk area 1 [1,26,27]. The current number of POU filters in the clinical complex of the University of Greifswald is 63 at round about 900 beds. The annual costs therefore add up to €60,000. Except for the ethic aspect the costs of a single case of a severe sepsis add up to €25,000 [28]. Only considering the rate of sepsis in very low birth neonates in our hospital the reduction from 2004 (46 %) to 2005 (11 %) underline the cost effectiveness.

As a result of these aspects the costs of the POU filters as an essential element of the preventive measures in high risk areas are more than justified even under economic considerations.

The next step is the definition of extended limiting values according to a risk assessment. Therefore we introduced the so-called warning, alert and worst case value. Especially the warning value is considered under aspects of primary prevention as one of the central requests on the WSP. The long term effect of this sub-set limit value is the avoidance of nosocomial infections. The alert and worst case values were established regarding to accidental and even situations of bioterrorism where immediate and more than ever efficient measures have to be predetermined.

In progression of the three years there is a trend from many re-inspections, flushing of the water system and installation of additional POU filters to heating up – even if only small amounts of *Legionella spp. *occur – and processing of aerators, as a reaction to a local contamination.

Summarizing all mentioned premises the WSP adapts the principles of primary prevention especially regarding to ethical aspects, in contrast to perform measures only as a response to increased quantities of nosocomial infections or even severe outbreaks. Impressive is the difference between 2004 and 2005, when the WSP and its defined measures were fully put into action with an awesome success compared to the outcome of former procedures.

To enforce basical ideas of WSP in developing countries for immunocompromised patients as well we suggest the following measures: boiled water for drinking and food preparation, use of medical devices (e.g. nebulizers), wound care especially burns no showering, no curtains for showers [29]. If boiling is not possible, sun exposition of water in plastic bottles has disinfecting effects [30].

## Conclusion

After the implementation of the WSP in all parts of the hospital the number of transgressions decreased continuously. Another argument for the efficacy of the WSP is the fact that there was no case of nosocomial Legionnaires' disease since the year 2004 although the institute of microbiology screened each case of pneumonia for Legionella.

The WSP offers the possibility starting measures in case of a contamination immediately by using previously defined actions according to a risk assessment. The advantage of a central recording and assessment of all results referring to the water quality combined with the possibility of a process control is an unalienable part around the efforts of the water safety plan. Beside the ethical aspect a WSP is cost effective.

## Competing interests

The author(s) declare that they have no competing interests.

## Authors' contributions

AD and AK carried out the study and drafted the manuscript. ME participated in the design of the study and helped to draft the manuscript. All authors read and approved the final manuscript.

## Pre-publication history

The pre-publication history for this paper can be accessed here:


